# Focal Cryoablation of the Prostate: Primary Treatment in 163 Patients With Localized Prostate Cancer

**DOI:** 10.7759/cureus.37172

**Published:** 2023-04-05

**Authors:** Anwar Khan, Ansar U Khan, Larry Siref, Michael Feloney

**Affiliations:** 1 Urology, Fremont Urology Health Center, Fremont, USA; 2 Urology, Creighton University School of Medicine, Omaha, USA

**Keywords:** targeted prostate cancer therapy, oncologic surveillance, cryotherapy prostate cancer, oncologic risk stratification, focal cryoablation prostate cancer

## Abstract

Objective

Whole gland treatment of the prostate has known efficacy in treating many grades of prostate cancer. However, it is often associated with increased morbidity, including erectile dysfunction and urinary incontinence. Focal ablative therapies, including focal cryoablation (FC), are utilized to minimize the risk of tumor progression and preserve erectile and urinary function. There is little to no consensus on whether intermediate or high-risk prostate cancer should be treated with focal therapy. However, there is a growing body of literature on the effectiveness of FC as an effective means of prostate cancer control. We characterize our experience with 163 patients who underwent FC with a median follow-up of 39 months (IQR: 24-60).

Methods

A 163-patient retrospective cohort underwent FC of the prostate at a single clinic by a physician from November 2008 to December 2020. Each of these T1c patients in this single-tail study was monitored for biochemical recurrence (BCR) and oncologic outcomes. American Society for Radiation Oncology (ASTRO) definition of BCR is three consecutive prostate-specific antigens (PSA) increases of more than 0.5 ng/mL or, along with the utilization of the Phoenix definition, a PSA greater than nadir by 2 ng/mL was used to define BCR. This study's primary endpoint includes BCR or biochemical disease-free survival rates. Secondary endpoints include patient side effects, such as measuring for urinary incontinence and outcomes of salvage treatments. Cox proportional hazard analyses defined univariate HRs and 95% CIs for pre-operative PSA (POPSA), Decipher, and Gleason Grade Groups (GGGs) to determine the prognostic impact of pathologic factors. Statistical analysis and BCR timeline analysis also included logistic regression and the Kaplan-Meier method, with significance considered at p < 0.05. Select focal cryotherapy patients were monitored utilizing genomic sequencing tests.

Results

Our cohort included 27 patients (16.5%) with D'Amico low, 115 patients (70.5%) with intermediate, and 23 patients (14.1%) with high-risk prostate cancers. One month after FC, a 73% reduction in PSA resulted in a median post-operative PSA of 1.39 ng/mL (IQR: 0.46-2.80 ng/mL). At five years, our cohort yielded biochemical disease-free recurrence rates of 78%, 74%, and 55% for low, intermediate, and high-grade cancers, respectively. Genetic risk stratification results showed very similar BCR rates to patients whose tissues did not undergo genomic testing, at 27%, 26%, and 46% for low, intermediate, and high-grade cancers, respectively. Log-rank tests to map for BCR and HRs for pathologic factors did not yield any statistically significant predictive results. Urinary incontinence and erectile dysfunction were reported in 1.8% and 3.1% of patients in the focal cohort.

Conclusions

Our results add to the expanding body of literature around the efficacy of focal ablative therapies in contrast to whole gland treatment. The definitive extent of FC's efficacy still remains to be explored, but our conclusions demonstrate favorable PSA kinetics at five years follow-up.

## Introduction

Prostate cancer is one of the most common types of cancer in men and is a leading pathology in the field of urology and oncology. Cryoablation of the prostate was initially used to treat clinically significant prostate cancer (PCa) in the 1960s with an open perineal approach and was often associated with high morbidity [1]. The procedure was reintroduced in 1993, using transrectal ultrasound to monitor the procedure in real time. American Urology Association (AUA) guidelines list treatments for clinically localized PCa as surveillance, prostatectomy, radiation therapy, cryosurgery, and high-intensity focused ultrasound therapy (HIFU). Treatment of the whole gland results in significant morbidity, including erectile dysfunction and urinary incontinence, adversely affecting the patient's quality of life [2]. The use of focal ablative therapies, including focal cryoablation (FC), has been explored to minimize the risk of tumor progression and preserve erectile and urinary function. Early reports of focal therapy for PCa were published as early as 2002; however, with the use of multi-parametric MRI, there has been a resurgence of interest in focal therapy [3]. There are limitations to focal therapy since PCa can be a multifocal disease, and there are opportunities to miss cancerous lesions during treatment. There is little to no consensus on whether intermediate or high-risk PCa should be treated with focal therapy. A more recent 2018 study suggested that only 38% of patients are possible candidates for focal therapy due to their histologic profile, and in this select group, a maximum of 4+3 Gleason Score, or Gleason Grade Group (GGG) 3, with or without foci under 4 mm at a score of Gleason 6 are possible candidates [4]. In other words, focal therapy has often only been recognized as a treatment for intermediate or low-grade cancers. We present our experience with 163 patients with low, intermediate, and high-grade cancers who underwent FC of PCa from November 2008 till December 2020, with a median follow-up of 39 months (Interquartile Range [IQR]: 24-60).

## Materials and methods

From 2008 to 2020, 163 T1c patients underwent focal, hemi-gland, or partial gland cryoablation of the prostate. These selected patients were offered FC as an alternative to more aggressive treatments such as total gland treatment with prostatectomy, radiation therapy, or total cryoablation. The criterion for selection in this patient cohort was men with confirmed organ-confined disease, any GGG, a negative metastatic result, and for whom preservation of erectile function and preventing urinary incontinence was a major goal. All patients underwent transrectal or a transperineal template saturated biopsy, and all samples were submitted in their entirety for standard pathologic analysis and histological mapping to determine GGG, pathological stage, and exact location of cancer. The primary outcomes of this study are biochemical recurrence (BCR). Secondary outcomes include patient side effects such as urinary incontinence and outcomes of salvage treatments. American Society for Radiation Oncology (ASTRO) defines biochemical recurrence as three consecutive prostate-specific antigen (PSA) increases of more than 0.5 ng/mL or, along with the utilization of the Phoenix definition, a PSA greater than nadir by 2 ng/mL [5]. Patients were seen for follow-ups every three months for the first year to monitor serum PSA for BCR. If their PSA was stable for a year, subsequent follow-up was every six months. Given that patients had BCR, recurrence was determined to be local or metastatic by subjecting patients to CT and bone scans if the primary tumor was poorly differentiated. If a salvage treatment was planned and the patient was not a candidate for radical prostatectomy, total cryoablation, or radiation, the patient was given androgen deprivation therapy (ADT). Kaplan-Meier curves and log-rank tests were mapped for BCR by GGG and Gleason Score. The failure timeline represents the months from the procedure in which the patient experienced BCR or the maximum amount of time from their last follow-up. To determine if histological groups were predictive of recurrence, log-rank tests for GGGs as they related to BCR were performed. Cox proportional hazard analyses defined univariate HRs and 95% CIs for pre-operative PSA (POPSA). Decipher (San Diego, CA) and GGGs were performed to determine the prognostic impact of pathologic factors. Each pathologic factor's first sub-group was utilized as the reference point for determining univariate HRs and CIs. For example, Decipher HRs were determined by setting low-risk (<0.45) patients as the reference to determine the HRs for medium (0.45-0.60) and high-risk (>0.60) Decipher categories. This was the same method utilized for GGG and POPSA. POPSAs were grouped into the following groups: <4.0 ng/mL, 4.0-10.0 ng/mL, 10.0-20.0 ng/mL, and 20.0+ ng/mL as PSA of 4.0 ng/mL has been the historic cut-off [6]. Decipher is a common genomic classifier that defines risk stratification as low, intermediate, or high for prostate cancer patients [7]. All statistical analysis was single-tailed and performed in Stata, with figures generated in R-Studio. Differences were considered significant if the p-value was < 0.05.

## Results

The average age of the patients who underwent focal cryotherapy was 65 years (range: 45-83 years), with an average serum PSA at the time of diagnosis at 6.55 ng/mL (IQR: 4.75-7.30 ng/mL) (Table 1).

**Table 1:** Summary of 163 patients who underwent focal cryoablation.

	IQR	Range	Median	Mean
Age	61-70	45-83	66	65
Pre-operative PSA (ng/mL)	4.75-7.30	1.46-24.3	5.86	6.55
Post-operative PSA (ng/mL)	0.46-2.80	0.01-6.7	1.39	1.84
Follow-up (Months)	24-60	-	39	-
Gleason Grade Group (GGG)	Count			
Gleason Grade Group 1 (Gleason 6)	27			
Gleason Grade Group 2 (Gleason 3+4)	89			
Gleason Grade Group 3 (Gleason 4+3)	26			
Gleason Grade Group 4 (Gleason 8)	15			
Gleason Grade Group 5 (Gleason 9/10)	6			
Total	163			

**Figure 1:**
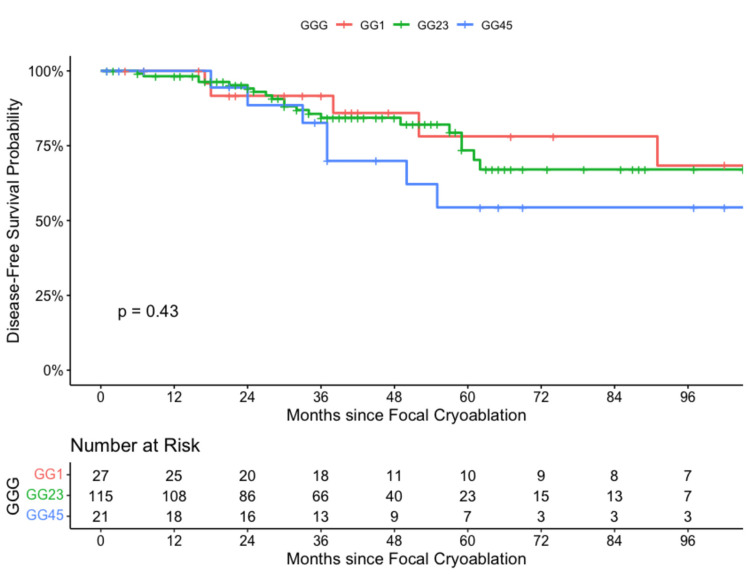
Focal cryoablation using Kaplan-Meier and Log-rank by GGG (2008-2020). GGG: Gleason Grade Group.

Biopsy types varied in these patient cohorts, with 28 patients undergoing transrectal prostate biopsy and the other 136 undergoing transperineal-mapping biopsy. There were 27 patients (16.5%) with D'Amico low, 115 patients (70.5%) with intermediate, and 23 patients (14.1%) with high-risk prostate cancers. One month after FC, a 73% reduction in PSA resulted in a median post-operative PSA of 1.39 ng/mL (IQR: 0.46-2.80 ng/mL). The median follow-up from the initial procedure was 39 months (IQR: 24-60). At 60 months, our clinical data on FC showed a local BCR of 22%, 26%, and 45% for low, intermediate, and high-grade cancers, respectively. In other words, this yields a biochemical disease-free survival rate of 78%, 74%, and 55% for low, intermediate, and high-grade cancers, respectively (Figure 1).

**Figure 2:**
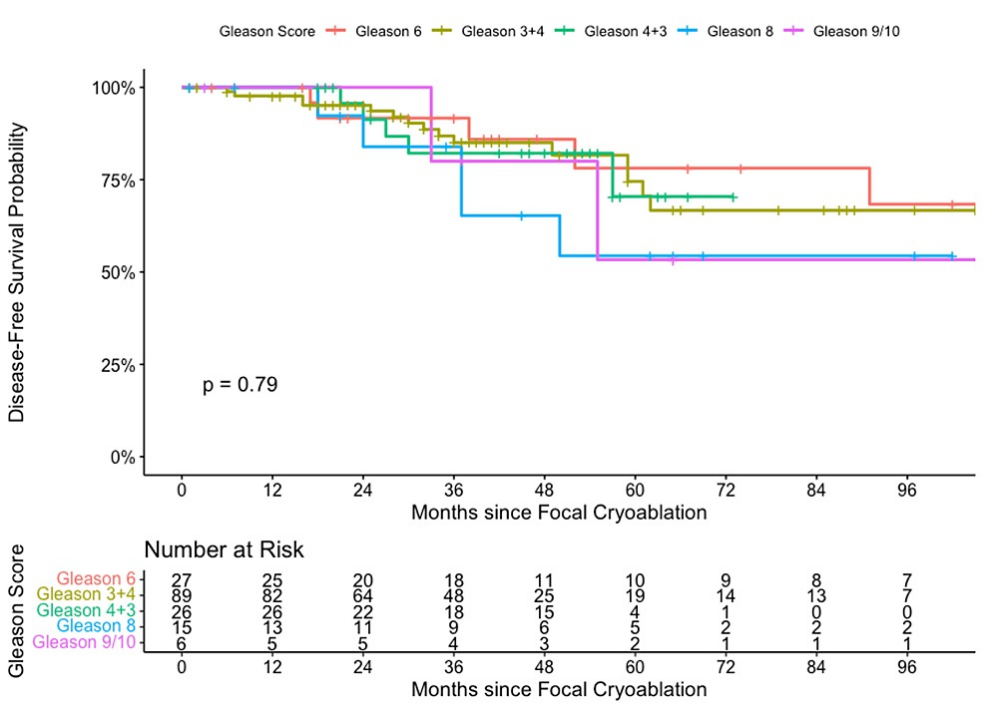
Focal cryoablation using Kaplan-Meier and Log-rank by Gleason Score (2008-2020).

Log-rank tests for Gleason Score PCa related to time-to-recurrence event yielded t (4) = 1.73, p = 0.79 (Figure 2).

**Figure 3:**
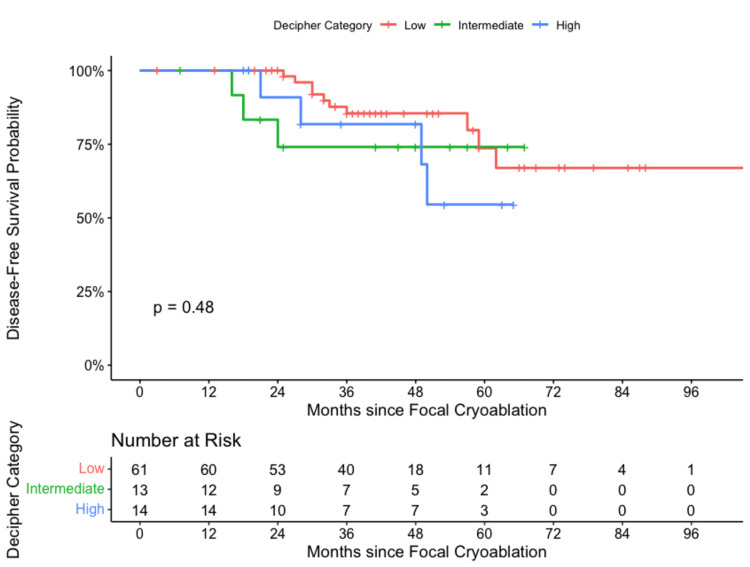
Focal cryoablation using Kaplan-Meier and Log-rank by Decipher score.

This indicates that there is no statistical difference between GGG in determining the expected BCR outcome. However, our Kaplan-Meier curves do trend towards higher-grade cancers failing earlier than lower-grade cancers. Thirty-two patients had local BCR in the FC cohort. After patients were confirmed to have localized BCR, salvage treatment included total cryoablation (12.5%), radiation therapy (9.4%), radical prostatectomy (15.6%), androgen deprivation therapy (ADT) (9.4%), multiple treatments (15.6%), and no further treatment (37.5%). The small patient subgroup that did not receive any further treatment was due to medical co-morbidities or the patients being lost to follow-up. In our sample, the loss to follow-up rate was 13.4%. Prostate biopsy tissue was subjected to genetic testing Decipher score analysis in 88 FC patients. These patients had viable tissue samples for genomic sequencing. Sixty-one (69.3%) patients had low risk (0-0.45), 13 (14.8%) had intermediate risk (0.45-0.60), and 14 (15.9%) had high risk (0.60-1.00) Decipher scores. At five years, BCR was 27%, 26%, and 46% for low, intermediate, and high-grade cancers, as per Decipher, respectively (Figure 3).

Univariate HRs for POPSA, Decipher (for those patients with available applicable biopsy tissues), and GGG, with each initial group in the set, were set as the reference for each pathological indicator. The POPSA low group (<4 ng/mL) vs. the highest POPSA group (>20 ng/mL) yielded an HR of 2.26 (95% CI: 0.59-153.84, p = 0.111). The lowest Decipher group (<0.45) vs. the highest Decipher group (>0.60) yielded an HR of 0.56 (95% CI: 0.55-5.50, p = 0.340). The lowest GGG group (Gleason 6) vs. the highest GGG group (Gleason 9/10) yielded an HR of 0.582 (CI: 95% 0.35-9.24, p = 0.487). None of these covariates or pathologic indicators predicted BCR at a statistically significant level (Table 2). 

**Table 2:** Predictors for biochemical recurrence and clinically significant prostate cancer recurrence for 163 patients in focal cryoablation cohort.

	Univariate	
Pre-Operative PSA	HR, CI: 95%	P-value
< 4 ng/mL vs 4-10 ng/mL	1.87, 0.88-47.91	0.065
< 4 ng/mL vs 10-20 ng/mL	0.71, 0.12-32.77	0.613
< 4 ng/mL vs >20 ng/mL	2.26, 0.59-153.84	0.111
Gleason Grade Groups (GGG)	HR, CI: 95%	P-value
GGG 1 vs. GGG 2	0.165, 0.43-3.26	0.751
GGG 1 vs. GGG 3	0.166, 0.33-4.13	0.795
GGG 1 vs. GGG 4	0.700, 0.58-6.96	0.27
GGG 1 vs. GGG 5	0.582, 0.35-9.24	0.487
Decipher (D)	HR, CI: 95%	P-value
D Low vs. D Intermediate	0.49, 0.46-5.91	0.445
D Low vs. D High	0.56, 0.56-5.50	0.34

In most men, below the average of 65 years, erectile function was maintained with or without 5-Phosphodiesterase inhibitors. In most cases, erectile dysfunction improved without pharmacologic intervention after nine to twelve months. Only 3.1% of patients reported erectile dysfunction in the FC cohort at any point in follow-up. Urinary incontinence was reported in 1.8% of patients in the focal cohort.

## Discussion

Eastham JA et al. (2022) characterize the current AUA/ASTRO Guidelines in their recent study and determine that prostate ablation should be utilized primarily in patients with intermediate-grade cancer. Clinicians should not suggest focal ablation for patients with high-risk PCa outside a clinical trial [8]. Other studies suggest that the efficacy of cryoablation is independent of DNA ploidy type or cellular differentiation [9]. Our biochemical disease-free survival rates of 78%, 74%, and 55% in low, intermediate, and high-grade cancers, respectively, add to the growing body of literature around treatment outcomes with primary cryoablation. Jones JS and Rewcastle JC (2008) focus on the impact of primary cryoablation for Gleason 8, 9, and 10 localized or high-grade PCa in 132 patients and reported satisfactory biochemical disease-free survival at five years of 64.4% by ASTRO definition [10]. Our results yield relatively similar biochemical disease-free-survival rates at 55% for high-grade cancers. Oishi M et al. (2019) define outcomes of hemi-gland cryoablation in low, intermediate, and high-grade PCa at five years [11]. This 160-patient cohort yielded 78%, 57%, and 67% biochemical failure-free for low, intermediate, and high-grade cancers, respectively. Our results show the same results for low-grade cancers and similar results to intermediate- and high-grade cancers in these two current, similar-sized, and retrospective cohort studies. Genetic testing scores trended towards PSA stability, with the highest-risk group having the highest chances of biochemical relapse. Additionally, our Decipher results showed similar BCR rates to our Kaplan-Meier method analysis of non-Decipher patients, at 27%, 26%, and 46% for low, intermediate, and high-grade cancers, respectively.

There is increasing interest in focal ablation of the prostate to avoid erectile dysfunction and urinary incontinence from prostatectomy or radiation toxicity to the rectum and bladder associated with whole gland treatment. Different modalities of targeted focal ablation are available, including HIFU, focal laser ablation, radiofrequency ablation, photodynamic therapy, and cryoablation. The technology for cryoablation has been present for many years; hence, it is a common modality for focal ablation of the prostate. It is, however, valuable to note the efficacy and growing body of literature around alternative focal therapy modalities. Guillaumier S et al. (2018) focused on five-year oncologic outcomes after HIFU and found 88% failure-free survival in treating non-metastatic PCa in a 625 multi-center cohort [12]. Musunuru HB et al. (2016) support active surveillance for low and intermediate-risk PCa with 15-year metastasis-free survival rates ranging from 64% to 94% [13]. Azzouzi AR et al. (2017) performed the first randomized clinical trial and assigned a 413 low-risk PCa patient cohort to photodynamic therapy or active surveillance [14]. This study found that at two years of follow-up, there were lower rates of histologic progression from photodynamic therapy (28%) rather than active surveillance (58%). The study additionally found that focal therapy reduced the rate of radical therapy for PCa from 53% to 24% in four years. There is a wide range of literature that discusses biochemical outcomes, prognostic indicators related to prostate ablation, and whether it is cryoablation or an alternative modality. Many reports support GGG, Decipher, and POPSA as accurate statistical predictors of BCR. However, our statistical analysis of HRs and log-rank tests yielded no statistically significant outcomes [7,11,15]. This is likely a result of low statistical power from our smaller cohort size. Aside from its statistically significant predictiveness, recent studies have also shown that Decipher can significantly reduce decision anxiety and patient-doctor conflict (18%) and can result in changes in salvage or adjuvant treatment decision-making (32%) [16]. For secondary outcomes, including urinary incontinence, our result of 1.8% was similar to that of other reports of 0%-3.0% [11,17]. Erectile dysfunction results varied in those same reports from 14% to 27%, compared to our result of 3.1% [11,17]. Limitations of our study include that this was a single-center, single-physician cohort study. Additionally, side effects, including erectile dysfunction, are often under-reported by patients. Further, monitoring the effectiveness of therapy ideally requires negative biopsies at 12 months. Most patients in this cohort did not wish to have repeat biopsies but preferred serial PSA monitoring at three-month intervals for one to two years. Limited post-operative biopsies (11.6% of the entire patient cohort) were performed to track the progression of oncologic outcomes for the entire cohort. While biopsy is preferred, serial PSA is a routinely preferred option for many patients, which has been utilized to capture expected cancer recurrence. Finally, patients being lost to follow-up or expiring due to other co-morbidities can have an effect on the follow-up data.

## Conclusions

Our results add to the expanding body of literature around the efficacy of focal ablative therapies in contrast to whole gland treatment. The extent of FC's efficacy still remains to be established, but our conclusions demonstrate favorable PSA kinetics at five years follow-up. Common complications such as erectile dysfunction and urinary incontinence were minimal but still present in the patient cohort. Additionally, this retrospective study can further quantify BCR for primary FC. Our experience was not limited to intermediate or low-grade PCa and provided insight into outcomes of FC for high-grade PCa. If further studies continue to add to the literature around primary and salvage FC with prolonged follow-up, FC could prove to be efficacious in PCa treatment with limited morbidity.

## References

[1] Gonder MJ, Soanes WA, Shulman S (1966). Cryosurgical treatment of the prostate. Invest Urol.

[2] Robinson JW, Donnelly BJ, Saliken JC, Weber BA, Ernst S, Rewcastle JC (2002). Quality of life and sexuality of men with prostate cancer 3 years after cryosurgery. Urology.

[3] Bahn DK, Lee F, Badalament R, Kumar A, Greski J, Chernick M (2002). Targeted cryoablation of the prostate: 7-year outcomes in the primary treatment of prostate cancer. Urology.

[4] Nassiri N, Chang E, Lieu P (2018). Focal therapy eligibility determined by magnetic resonance imaging/ultrasound fusion biopsy. J Urol.

[5] Roach M 3rd, Hanks G, Thames H Jr, Schellhammer P, Shipley WU, Sokol GH, Sandler H (2006). Defining biochemical failure following radiotherapy with or without hormonal therapy in men with clinically localized prostate cancer: recommendations of the RTOG-ASTRO Phoenix Consensus Conference. Int J Radiat Oncol Biol Phys.

[6] Catalona WJ, Smith DS, Ratliff TL (1991). Measurement of prostate-specific antigen in serum as a screening test for prostate cancer. N Engl J Med.

[7] Dalela D, Löppenberg B, Sood A, Sammon J, Abdollah F (2016). Contemporary role of the Decipher® test in prostate cancer management: current practice and future perspectives. Rev Urol.

[8] Eastham JA, Auffenberg GB, Barocas DA (2022). Clinically localized prostate cancer: AUA/ASTRO guideline, Part I: Introduction, Risk Assessment, Staging, and Risk-Based Management. J Urol.

[9] Bahn DK, Silverman P, Lee F Sr, Badalament R, Bahn ED, Rewcastle JC (2004). In treating localized prostate cancer the efficacy of cryoablation is independent of DNA ploidy type. Technol Cancer Res Treat.

[10] Jones JS, Rewcastle JC (2008). Primary cryoablation for Gleason 8, 9, or 10 localized prostate cancer: Biochemical and local control outcomes from the Cryo OnLine database registry. Indian J Urol.

[11] Oishi M, Gill IS, Tafuri A (2019). Hemigland cryoablation of localized low, intermediate and high risk prostate cancer: oncologic and functional outcomes at 5 years. J Urol.

[12] Guillaumier S, Peters M, Arya M (2018). A multicentre study of 5-year outcomes following focal therapy in treating clinically significant nonmetastatic prostate cancer. Eur Urol.

[13] Musunuru HB, Yamamoto T, Klotz L (2016). Active surveillance for intermediate risk prostate cancer: survival outcomes in the Sunnybrook experience. J Urol.

[14] Azzouzi AR, Vincendeau S, Barret E (2017). Padeliporfin vascular-targeted photodynamic therapy versus active surveillance in men with low-risk prostate cancer (CLIN1001 PCM301): an open-label, phase 3, randomised controlled trial. Lancet Oncol.

[15] Liu YY, Chiang PH, Chuang YC, Lee WC, Cheng YT, Wang HJ (2015). Predictors of prostate-specific antigen biochemical recurrence in patients undergoing primary whole-gland prostate cryoablation. Ann Surg Oncol.

[16] Gore JL, du Plessis M, Santiago-Jiménez M (2017). Decipher test impacts decision making among patients considering adjuvant and salvage treatment after radical prostatectomy: interim results from the Multicenter Prospective PRO-IMPACT study. Cancer.

[17] Bahn D, de Castro Abreu AL, Gill IS (2012). Focal cryotherapy for clinically unilateral, low-intermediate risk prostate cancer in 73 men with a median follow-up of 3.7 years. Eur Urol.

